# The Predictive Performance of the STOP-Bang Questionnaire in Obstructive Sleep Apnea Screening of Obese Population at Sleep Clinical Setting

**DOI:** 10.7759/cureus.6498

**Published:** 2019-12-29

**Authors:** Haluk Mergen, Burak Altındağ, Zeynep Zeren Uçar, Işıl Karasu Kılıçaslan

**Affiliations:** 1 Family Medicine, University of Health Sciences, Izmir Tepecik Health Practice & Research Center, Izmir, TUR; 2 Sleep Medicine, University of Health Sciences, Izmir Dr. Suat Seren Health Practice & Research Center, Izmir, TUR

**Keywords:** : obstructive sleep apnea, obesity, stop-bang questionnaires, sleep apnea screening

## Abstract

Background

The prevalence of obstructive sleep apnea (OSA) is high in the obese population. In this study, it was aimed to fulfill the STOP-Bang questionnaire which is a concise and easy-to-use questionnaire for OSA screening in obese patients.

Materials & methods

This is a retrospective study where the patients, who planned polysomnography, were referred to sleep clinic. Patients were screened for OSA by the STOP-Bang questionnaire. Laboratory polysomnography was performed in 275 patients. Patients with BMI ≥ 30 were taken into study. The screening test was evaluated by three different risk analysis such as, a STOP score, a STOP-Bang score and a modified STOP-Bang score. The predictive parameters (sensitivity, specificity, and positive and negative predictive values) for alternative scoring models in obese patients were analyzed.

Results

In 217 obese patients, a STOP score cutoff of 3 and a STOP-Bang score cutoff of 4 provides a better balance of sensitivity and specificity for all OSA (apnea-hypopnea index [AHI] ≥ 5). The STOP questionnaire revealed a sensitivity of 87.9% and a positive predictive value of 99.5% for patients with all OSA (p: 0.005). The STOP-Bang scoring model revealed a sensitivity of 95.3% and a positive predictive value of 99.5% for patients with all OSA (p < 0.001). The modified STOP-Bang scoring revealed a sensitivity of 95.8% and a positive predictive value of 99.5% for patients with all OSA (p < 0.001). The area under the curve of the STOP-Bang for identifying mild, moderate and severe OSA was 0.581, 0.652 and 0.675, respectively. Whereas according to the STOP-Bang, all morbid obese patients (obesity class III, n: 47) were at high risk of OSA.

Conclusion

This study suggests that the STOP-Bang questionnaire for obstructive sleep apnea screening in obese patients is a high sensitivity and appropriate screening test.

## Introduction

The prevalence of obstructive sleep apnea (OSA) was found to range from 3.1% to 7.5% in males and from 2.1% to 4.5% in females in studies conducted in different societies [1-6]. The most common symptoms of OSA are snoring, probable apnea, excessive daytime sleepiness, waking up with choking sensation, insomnia [7-9]. Although many symptoms occur during the day and night, most of the patients are not aware of their illness [10].

OSA is a treatable disease. However, overnight polysomnography (PSG), which is the gold standard for the diagnosis of OSA, is a difficult diagnostic method to reach [11]. Polysomnography is not available in many centers and the appointment times are long because it is a time consuming process [12]. Similar to many diseases, OSA has also developed questionnaires based on clinical and laboratory findings. In the questionnaires, sleep quality, symptoms of sleep disturbance, risk factors of sleep disturbance and possible complications related to sleep problems are questioned.

According to the World Health Organization in 2016, more than 1.9 billion adults, 18 years and older, were overweight. Of these, over 650 million were obese. The prevalence of OSA is high in the obese population. OSA risk was increased 8-12 times in cases with BMI > 29 [13]. In this study, it was aimed to apply the STOP-Bang questionnaire among obese patients which is not most studied.

## Materials and methods

The study was conducted after the approval of the Ministry of Health Sciences, Izmir Tepecik Education and Research Hospital Ethics Committee with the decision of 1 date and number 1. Between January 2017 and December 2017, adult patients, who applied to Sleep Disorders Clinic, Dr. Suat Seren Chest Diseases and Surgery Training and Research Hospital, because of obstructive sleep apnoea syndrome (OSAS) doubt and performed PSG, were included in the study. Patients with BMI 30-34.9 were classified as class 1 obese and patients with BMI 35-39.9 were classified as class 2 obese and patients with BMI ≥ 40 were classified as class 3 (morbid obese). Patients with a BMI < 30 were not included in the study. Before the STOP-Bang questionnaire was filled, patients were informed and their consent was obtained.

STOP-Bang survey

The STOP-Bang questionnaire consists of eight questions. These questions are answered as “yes” or “no”. The STOP questionnaire consists of the first four questions and is considered high risk for OSA and a “yes” response to fewer questions is given with at least two “yes” answers. The STOP-Bang questionnaire was evaluated by two different methods. If at least three of the questions were given a “yes” answer at the first assessment, a high risk for OSA and a “yes” response to lesser questions were considered low risk for OSA. The second assessment was made by the University Health Network in 2014 and used low intermediate-high risk scoring criteria for OSA with various criteria. These criteria are given in Table 1.

**Table 1:** Updated STOP-Bang Questionnaire Scoring Criteria OSA: Obstructive sleep apnea

Low risk of OSA: “Yes” to 0-2 questions
Intermediate risk of OSA: “Yes” to 3-4 questions
High risk of OSA: “Yes” to 5-8 questions
or “Yes” to 2 or more of 4 STOP questions + male gender
or “Yes” to 2 or more of 4 STOP questions + BMI > 35 kg/m^2^
or “Yes” to 2 or more of 4 STOP questions + neck circumference (17”/43 cm in male, 16”/41 cm in female

Polysomnography

In sleep laboratory, sleep structure (electroencephalogram, electrooculogram and submental electromyogram), breathing (thoracic and abdominal measurements), limb movements, nasal airflow and snoring severity were recorded. Percutaneous oxygen saturation (SpO2) and transcutaneous pulse oximetry were used for heart rate measurement. Based on the criteria of the American Academy of Sleep Medicine, the apnea-hypopnea index (AHI) value was used to determine the severity of OSA. Apnea was defined as a persistent airflow of at least 10 seconds. Periods in which airflow decreased by more than 30% and SpO2 decreased by more than 4% for at least 10 seconds were defined as hypopnea. The total number of apnea and hypopnea events per hour was also calculated as AHI. Thus, if the AHI value is ≤5, the patient was considered not to have OSA. The AHI score was defined as mild OSA of 5-15, moderate OSA of 15-30, and severe OSA of >30.

Statistical analysis

The data was saved by creating a Microsoft Excel file. Analyses of the data were made with the program “SPSS for Windows, Version 22.0” (IBM Corp., Armonk, NY). Descriptive statistics, percent for nominal variables, mean ± standard deviation or interquartile range for continuous variables are used. The questions of demographic data and STOP-Bang questionnaire were first divided into three groups according to obesity classification and then divided into risk groups according to three different questionnaire scores and evaluated by Pearson analysis method. The predictive parameters (sensitivity, specificity, and positive and negative predictive values) for alternative scoring models in obese patients were analyzed. Area under the curve for each score was STOP-Bang questionnaire calculated. A p-value of <0.05 was considered for statistical significance in the evaluations.

## Results

A total of 275 obese patients referred to the Working Sleep Disease Polyclinic were included. However, 217 patients were considered eligible for the study. Gender, age, BMI, neck circumference, cigarette and alcohol use, and additional diseases are included in Table 2 according to obesity classification. According to the classification made in the study, there was a significant relationship between neck circumference and BMI (p < 0.05). In addition, there is a significant relationship between diabetes, asthma and hypothyroidism and BMI in patients who have additional diseases (p < 0.05).

**Table 2:** Demographic and anthropometric data of the obese patients Data are depicted as number (percentage), mean ± SD, or median (interquartile range). CAD: Coronary artery disease; CHF: Congestive heart failure; COPD: Chronic obstructive pulmonary disease.

	Obesity Class I	Obesity Class II	Obesity Class III (Morbidly)	Total
n	104	66	47	217
Gender, n (F/M)	21/83	19/47	19/28	59/158
Age, years	50.5 ± 11	50.3 ± 9	49.7 ± 11	50.2 ± 10
BMI, kg/m^2^	32 ± 1	36.8 ± 1	43.4 ± 2	35.9 ± 4
Neck, cm	42.5 ± 3	43.1 ± 3	44.4 ± 3	43.1 ± 3
Smoke, n (%)	39 (37.5)	23 (34.8)	13 (27.7)	75 (34.6)
Alcohol, n (%)	21 (20,2)	9 (13.6)	3 (6.4)	33 (15.2)
Comorbidities				
CAD, n (%)	15 (14.4)	14 (21.2)	9 (19.1)	38 (17.5)
CHF, n (%)	2 (1.9)	1 (1.5)	3 (6.4)	6 (2.8)
Arrhythmia, n (%)	11 (10.6)	4 (6.1)	4 (8.5)	19 (8.8)
Hypertension, n (%)	41 (39.4)	35 (53)	25 (53.2)	101 (46.5)
Diabetes, n (%)	19 (18.3)	26 (39.4)	24 (51.1)	69 (31.8)
Asthma, n (%)	9 (8.7)	10 (15.2)	12 (25.5)	31 (14.3)
COPD, n (%)	9 (8.7)	9 (13.6)	6 (12.8)	24 (11.1)
Hypothyroidism, n (%)	7 (6.7)	4 (6.1)	9 (19.1)	20 (9.2)

According to the obesity classification of patients, AHI, severity of OSA and alternative scoring models of STOP-Bang are shown in Table 3. There was no statistically significant relationship between BMI, AHI, OSA and all three alternative scoring models of the patients (p > 0.05).

**Table 3:** Polysomnography results: AHI, OSA severity AHI: Apnea-hypopnea index; OSA: Obstructive sleep apnea.

	Obesity Class I	Obesity Class II	Obesity Class III (Morbidly)	Total
AHI median (25-75th)	39.6 (21-61)	48.1 (24-64)	53.3 (23.4-78.5)	45.6 (22-64)
Severity of OSA, n (%)				
No OSA	2 (1.9)	0 (0)	1 (2.1)	3 (1.4)
Mild OSA	16 (15.4)	7 (10.6)	2 (4.3)	25 (11.5)
Moderate OSA	20 (19.2)	16 (24.2)	9 (19.1)	45 (20.7)
Severe OSA	66 (63.5)	43 (65.2)	35 (74.5)	144 (66.4)

According to AHI > 5 cut-off, gender (p < 0.001), age (p = 0.041), coronary artery disease (p = 0.001), congestive heart failure (p < 0.001), arrhythmia (p < 0.001), hypertension (p < 0.001), hypothyroidism (p < 0.001), asthma (p < 0.001), chronic obstructive pulmonary disease (p < 0.001), STOP (p < 0.001), STOP-BANG (p < 0.001), modified STOP-BANG (p < 0.001) scores were found statistically significant.

The STOP questionnaire revealed a sensitivity of 87.9% and a positive predictive value of 99.5% for patients with all OSA (p: 0.005). The STOP-Bang scoring model revealed a sensitivity of 95.3% and a positive predictive value of 99.5% for patients with all OSA (p < 0.001). The modified STOP-Bang scoring revealed a sensitivity of 95.8% and a positive predictive value of 99.5% for patients with all OSA (p < 0.001). Area under the curve of the STOP-Bang for identifying mild, moderate and severe OSA was 0.581, 0.652, and 0.675, respectively. Whereas according to the STOP-Bang, all morbid obese patients (obesity class III, n: 47) were at high risk of OSA. In 217 obese patients, a STOP score cutoff of 3 and a STOP-Bang score cutoff of 4 provides a better balance of sensitivity and specificity for all OSA (AHI ≥ 5). In addition, the predictive parameters between the questions in the questionnaire and the OSA diagnoses are listed in Table 4. Table 5 shows the predictive parameters according to the OSA severity.

**Table 4:** STOP-Bang questionnaire data of the obese patients Data are depicted as number (percentage), mean ± SD, or median (interquartile range). LR: Low risk; IR: Intermediate risk; HR: High risk; STOP: Snoring, tiredness, observed apnea; STOP-Bang: Snoring, tiredness, observed apnea, blood pressure, body mass index, age, neck circumference and gender.

Positive response of STOP-Bang, n (%)	Obesity Class I	Obesity Class II	Obesity Class III (Morbidly)	Total
Q1 (Snoring)	97 (93.3)	63 (95.5)	44 (93.6)	204 (94)
Q2 (Tired or sleepy)	93 (89.4)	59 (89.4)	40 (85.1)	192 (88.5)
Q3 (Observed apnea)	94 (90.4)	58 (87.9)	41 (87.2)	193 (88.9)
Q4 (High blood pressure)	46 (44.2)	39 (59.1)	24 (51.1)	109 (50.2)
Score BMI (>35 kg/m^2^)	0 (0)	52 (78.8)	47 (100)	99 (45.6)
Score Age (>50 years)	53 (51)	33 (50)	20 (42.6)	106 (48.8)
Score Neck (>40 cm)	39 (37.5)	40 (60.6)	36 (76.6)	115 (53)
Score Gender	83 (79.8)	47 (71.2)	28 (59.6)	158 (72.8)
Alternative scoring models of STOP-Bang questionnaire				
STOP, n (LR-HR)	15-89	7-59	6-41	28-189
STOP-Bang, n (LR-HR)	9-95	3-63	0-47	12-205
STOP-Bang modifying, n (LR-IR-HR)	2-3-99	1-2-63	0-3-44	3-8-206

**Table 5:** Predictive parameters PPV: Positive predictive value; NPV: Negative predictive value; OSA: Obstructive sleep apnea.

All OSA (AHI > 5)	Sensitivity (%)	Specificity (%)	PPV (%)	NPV (%)
Q1 (Snoring)	94.4%	33.3%	99.0%	7.7%
Q2 (Tired or sleepy)	88.3%	0.0%	98.4%	0.0%
Q3 (Observed apnea)	89.7%	66.7%	99.5%	8.3%
Q4 (High blood pressure)	50.5%	66.7%	99.1%	1.9%
Score BMI (>35 kg/m^2^)	45.8%	66.7%	99.0%	1.7%
Score Age (>50 years)	49.1%	66.7%	99.1%	1.8%
Score Neck (>40 cm)	53.7%	100.0%	100.0%	2.9%
Score Gender	73.8%	100.0%	100.0%	5.1%
Moderate/Severe OSA (AHI > 15)				
Q1 (Snoring)	95.2%	14.3%	88.2%	30.8%
Q2 (Tired or sleepy)	87.3%	3.6%	85.9%	4.0%
Q3 (Observed apnea)	91.5%	28.6%	89.6%	33.3%
Q4 (High blood pressure)	50.8%	53.6%	88.1%	13.9%
Score BMI (>35 kg/m^2^)	49.2%	78.6%	93.9%	18.6%
Score Age (>50 years)	49.2%	53.6%	87.7%	13.5%
Score Neck (>40 cm)	54.0%	53.6%	88.7%	14.7%
Score Gender	73.5%	32.1%	88.0%	15.3%
Severe OSA (AHI > 30)				
Q1 (Snoring)	96.5%	11.0%	68.1%	61.5%
Q2 (Tired or sleepy)	84.0%	2.7%	63.0%	8.0%
Q3 (Observed apnea)	93.1%	19.2%	69.4%	58.3%
Q4 (High blood pressure)	50.7%	50.7%	67.0%	34.3%
Score BMI (>35 kg/m^2^)	50.7%	64.4%	73.7%	39.8%
Score Age (>50 years)	50.0%	53.4%	67.9%	35.1%
Score Neck (>40 cm)	56.3%	53.4%	70.4%	38.2%
Score Gender	77.8%	37.0%	70.9%	45.8%

## Discussion

In the study, BMI, neck circumference, hypertension, diabetes, mean O_2_ saturation, BMI score, neck score and gender scores were found statistically significant among obesity classes like in the study of Chung et al.. Stop-Bang scores were found statistically significant with hypertensive patients, absence of OSA, severe OSA, AHI at greater than 5, 15 and 30, Q1 (snoring), Q3 (observed apnea), Q4 (high blood pressure), BMI score, neck score. The STOP-Bang questionnaire was associated with high sensitivity and PPV at AHI > 5 and AHI > 15 rather than AHI > 30.

Studies indicate high incidence of coronary artery disease, hypertension, cerebrovascular accidents, gastroesophageal reflux, congestive heart failure, myocardial infarction with OSA [14,15]. Hypertension was found statistically significant among high risk patients in our study too. The sensitivity varied from 76% to 96% and specificity varied from 70% to 93% [16-18]. Our sensitivity and specificity values are lower than those, perhaps our patients are solely obese patients. BMI, age (>50 years old), neck circumference, and gender score have very high PPV for OSA (nearly 100% at AHI > 5). Specificity was found lower as in Acar et al. study [19].

In a study, STOP-Bang questionnaire was used among surgical patients (27.5%); in another study, 41.5% of patients found under high risk [20]. In our study, this ratio was 94.4%. In a study including bariatric surgery patients, high-risk patients were 80% which is similar to our study [21].

In comparison of AHI > 5 cut-off, gender, age, hypertension were found statistically significant as in Chung et al. and Acar et al. study [20]. The STOP-Bang score cutoff of 4 provides a better balance of sensitivity and specificity for all OSA (AHI ≥ 5), it is similar with the Chung et al.’s study [20,22].

In a recent study where OSA was evaluated among pregnant women with obesity during the second trimester of pregnancy, predictability of OSA is increased with high scores in STOP-Bang scores (score < 3: <10%, score 6: <3-68%). Best fitted model of STOP-Bang is the model followed with Q1 (snoring). Predictability was 5% without snoring and 26.5% with snoring. In our trial also, STOP-Bang score was found significant with Q1 (snoring). In that study, hypertension prevalence was found low despite other studies reported hypertension 8-50%. In our study, hypertension was found statistically significant at AHI > 5 cut-off with gender and age [23].

In a new study, STOP-Bang and polysomnography were performed among 541 Koreans where the usefulness of the items constituting is explored: SOPBAG (when T and A, tiredness and age, are excluded) had a better area under the curve (AUC) of 0.811, sensitivity 89.1% and specificity 57.4% than AUC of 0.809, sensitivity 71.7% and specificity 77.9% with STOP-Bang for Koreans. But the statistical significance between two AUC values needs to be further evaluated. This study's AUCs are better than our study's AUCs defining mild, moderate and severe OSA as 0.581, 0.652, and 0.675, respectively [24].

In a study fulfilled among hemodialysis patients, sleep-disordered breathing (SDB) is very common among dialysis patients (70%). STOPBANG-fixed 89% of patients had SDB. Polysomnography-fixed 86% had obstructive sleep apnoea and median AHI was 34.5/h. Oximetry and STOPBANG scores were positively correlated (r = 0.62, p = 0.0001; r = 0.48, p < 0.0001). Neck circumference (OR: 1.20; 95% CI: 1.07-1.34; p = 0.02) and haemoglobin (OR: 0.93; 95% CI: 0.88-0.97; p = 0.003) were found independently associated with SDB. In our study too, neck scores found statistically significant with STOPBANG scores [25].

## Conclusions

The STOP-Bang and STOP questionnaires are found as a valid scale among Turkish obese OSA patients in this study. The STOP-Bang scoring model revealed a sensitivity of 95.3% and a positive predictive value of 99.5%. The STOP questionnaire revealed a sensitivity of 87.9% and a positive predictive value of 99.5%. The modified STOP-Bang scoring revealed a sensitivity of 95.8% and a positive predictive value of 99.5%, for patients with all OSA. These questionnaires are useful and cost-effective tools that can be applied to all OSA patients rather than polysomnography and oxymetry. There are very few studies performed among obese patients, this study is one of them. Our limitation was that we had fewer number of patients with Obesity Class III (n = 47) rather than Obesity Class I and II patients. Higher number of obese OSA patients could reveal better results.
